# Emerging Oral Therapies for the Treatment of Psoriasis: A Review of Pipeline Agents

**DOI:** 10.3390/pharmaceutics16010111

**Published:** 2024-01-15

**Authors:** Anastasia Drakos, Tiago Torres, Ronald Vender

**Affiliations:** 1 Faculty of Medicine, University of Ottawa, Ottawa, ON K1H 8M5, Canada; adrak095@uottawa.ca; 2 Instituto de Ciências Biomédicas Abel Salazar, University of Porto, 4050-313 Porto, Portugal; torres.tiago@outlook.com; 3 Department of Dermatology, Centro Hospitalar de Santo António, 4099-001 Porto, Portugal; 4 Dermatrials Research Inc. & Venderm Consulting, Hamilton, ON L8N 1Y2, Canada; 5 Department of Medicine, McMaster University, Hamilton, ON L8N 3Z5, Canada

**Keywords:** psoriasis, small molecules, oral microbials, interleukin (IL)-23, IL-17, phosphodiesterase-4, Janus kinase, A3 adenosine receptor

## Abstract

The introduction of biologic agents for the treatment of psoriasis has revolutionized the current treatment landscape, targeting cytokines in the interleukin (IL)-23/IL-17 pathway and demonstrating strong efficacy and safety profiles in clinical trials. These agents however are costly, are associated with a risk of immunogenicity, and require administration by intravenous or subcutaneous injection, limiting their use among patients. Oral therapies, specifically small molecule and microbiome therapeutics, have the potential to be more convenient and cost-effective agents for patients and have been a focus of development in recent years, with few targeted oral medications available for the disease. In this manuscript, we review pipeline oral therapies for psoriasis identified through a search of ClinicalTrials.gov (30 June 2022–1 October 2023). Available preclinical and clinical trial data on each therapeutic agent are discussed. Small molecules under development include tumor necrosis factor inhibitors, IL-23 inhibitors, IL-17 inhibitors, phosphodiesterase-4 inhibitors, Janus kinase inhibitors, A3 adenosine receptor agonists, and sphingosine-1-phosphate receptor 1 agonists, several of which are entering phase III trials. Oral microbials have also demonstrated success in early phase studies. As new oral therapies emerge for the treatment of psoriasis, real-world data and comparative trials are needed to better inform their use among patients.

## 1. Introduction

Psoriasis is a chronic, immune-mediated inflammatory condition characterized by erythematous, scaling plaques on the skin [1,2]. The disease affects 2–3% of the world’s population and is associated with several comorbidities including cardiovascular disease, inflammatory bowel disease, and psoriatic arthritis [3,4,5]. Treatment requires an individualized approach, as mild cases are often managed with topical therapies, while moderate-to-severe disease (>5–10% body surface area) may require the addition of systemic therapies [6,7].

In recent years, considerable focus has been placed on improving our understanding of the disease as a means of discovering new targets for treatment. Although the pathogenesis of psoriasis is incompletely understood, current evidence suggests that psoriasis inflammation results from a complex interplay between the immune system, autoantigens, and environmental stimuli [1,2]. In patients with a genetic predisposition for the disease, triggers such as infection, skin trauma, or medications can activate keratinocytes to produce antimicrobial peptides, interleukin (IL)-1 family cytokines, and chemokines. These peptides stimulate plasmacytoid dendritic cells to secrete interferon (IFN)-α, promoting the downstream activation of myeloid dendritic cells. Once activated, myeloid dendritic cells act as antigen-presenting cells, secreting IL-12 and IL-23 and modulating the differentiation of T helper (Th) 1 and Th17 cell lines. Th17 is the predominant T-cell type driving psoriasis inflammation, secreting cytokines such as IL-17, IL-22, and TNF, that promote keratinocyte proliferation, angiogenesis, and epidermal hyperplasia. This drives a feed-forward mechanism that leads to the formation of psoriasis plaques [1,2].

Recognizing the role of the IL-23/IL-17 axis as the main pathogenic pathway in psoriasis has led to the development of several psoriasis-specific therapies. Specifically, several new biologic agents have been approved, with IL-12/23, IL-23, IL-17, and TNF inhibitors demonstrating superior efficacy to conventional systemic therapies in clinical trials and redefining treatment targets for moderate-to-severe disease [8,9,10]. These agents, however, are costly, are associated with a risk of immunogenicity, and require administration by intravenous or subcutaneous injection, justifying the search for further therapeutic solutions [11,12].

A shift towards the development of more effective oral therapies has been seen in the psoriasis treatment pipeline [13]. Specifically, small molecules are the subject of recent research, with these agents carrying a low molecular weight (<1 kDa) and the ability to cross cell membranes, directly blocking intracellular signaling pathways [13,14]. Compared to biologics, these agents are easier to synthesize, less expensive to produce, and may be administered orally or topically, improving convenience and quality of life for patients [13,14]. Oral microbials are also under investigation, however research is still preliminary [15]. Current small molecule options for psoriasis include apremilast, a phosphodiesterase-4 (PDE4) inhibitor, and deucravacitinib, a recently approved tyrosine kinase 2 (TYK2) inhibitor [16,17]. Apremilast, though commonly used in clinical practice, is associated with limited efficacy and gastrointestinal adverse events [18,19]. Deucravacitinib has been demonstrated to be more effective than apremilast in clinical trials, however, real-world data are lacking, and it remains unclear how this agent will perform against existing biologics [20,21]. 

New oral therapies are needed for the treatment of psoriasis. Here, we review oral agents in clinical development for the disease, highlighting their mechanism of action and summarizing the most recent clinical trial data, with the purpose of serving as an update for clinicians.

## 2. Methods

ClinicalTrials.gov was searched using the term “Psoriasis” for recently completed, ongoing, or newly initiated trials investigating pipeline oral therapies for the disease from 30 June 2022–1 October 2023. Agents in phase I, phase II, or phase III development were included. Literature searches on each identified agent were conducted using PubMed, searching “Drug Name” OR “Alternative Name.” Additional searches for abstracts, press releases, and presentations were performed, and findings were considered for inclusion.

## 3. Tumor Necrosis Factor Inhibitors

TNF is a pleiotropic cytokine involved in the pathogenesis of several autoimmune and inflammatory diseases [22,23]. It exists in two biologically active forms: a membrane-bound form (mTNF) and a soluble protein form (sTNF); the latter is released via proteolytic cleavage of the former by TNF converting enzyme [24]. Both mTNF and sTNF are active in their trimeric form and signal through two cognate receptors, TNFR1 and TNFR2 [25,26,27]. TNFR1 is ubiquitously expressed and is responsible for most of the biological effects of TNF, activating the nuclear factor kappa B (NF-κB) and mitogen-activated protein kinase (MAPK) pathways and promoting a proinflammatory response [27,28]. TNFR2 only responds to mTNF and is restricted to immune cells, endothelial cells, and glia, the activation of which promotes cell proliferation, survival, and lineage stability [29,30].

In patients with psoriasis, TNF is overexpressed, amplifying psoriasis inflammation through multiple pathways [31]. Specifically, TNF recruits lymphocytes to the site of inflammation and facilitates their entry into lesional skin, inducing the expression of adhesion molecules on vascular endothelial cells [32]. TNF also activates dermal dendritic cells and macrophages and acts alongside IL-17 to promote the differentiation and proliferation of keratinocytes, driving the formation of psoriasis plaques [32]. Current biologic agents targeting this pathway inhibit both mTNF and sTNF and remain important therapies for the treatment of disease [33,34]. Oral small molecules with a similar mechanism of action are currently under investigation.

SAR441566 (Sanofi) is a small molecule inhibitor of the TNF cytokine in early phase development for psoriasis [35,36]. It acts by stabilizing the asymmetrical form of the soluble TNF trimer, preventing its interaction with TNFR1 and inhibiting proinflammatory cytokine production [35]. Results from a phase I, double-blind, placebo-controlled, randomized trial (NCT05453942) were recently reported [36]. The trial enrolled 38 participants with mild-to-moderate psoriasis, and participants were randomized 2:1 to receive oral SAR441566 or placebo twice daily for 4 weeks. Fifty-eight percent of participants in the treatment group achieved a one-point reduction in the Investigator Global Assessment (IGA) score at 4 weeks compared to those who received the placebo (0%, *p* = 0.003). Mean changes in Target Lesion Severity (TLS) and Psoriasis Area and Severity Index (PASI) scores favoured the treatment group, with statistically significant reductions seen at 4 weeks. The drug was well tolerated by participants with no serious adverse events reported [36]. SAR441566 is currently being investigated in a phase II trial for psoriasis, with the PASI 75 response at 12 weeks defined as the study’s primary endpoint (NCT06073119).

## 4. Interleukin-23 Inhibitors

IL-23 is a major regulator of the Th17 pathway [37]. It is produced by myeloid dendritic cells and macrophages, and upon production drives the differentiation of naïve T helper cells to Th17 lymphocytes, stimulating these cells to secrete proinflammatory cytokines such as IL-12, IL-23, IL-17, and TNF [38,39,40]. Traditionally, agents that inhibit this pathway target one of the cytokine’s subunits (p19 or p40) and require an intravenous or subcutaneous route of administration [40]. Oral small molecules with a similar mechanism of action are currently under investigation.

JNJ-2113 (formerly PN-235; Johnson & Johnson Innovative Medicine, Cambridge, MA, USA), is an orally available peptide that competitively binds to the IL-23 receptor [41]. The agent potently blocks downstream cytokine signaling, reporting similar efficacy to an IL-23 antibody in imiquimod-induced psoriasis-like mouse models of inflammation, reducing skin thickness and downstream cytokine production [41]. In a phase IIb clinical trial (FRONTIER 1; NCT05223868), treatment with three once daily (25 mg, 50 mg, 100 mg) and two twice daily (25 mg, 100 mg) doses of JNJ-2113 led to significant improvements in PASI 75 response at 16 weeks compared to placebo [42]. Trends in PASI 90 and PASI 100 responses were consistent with the primary outcome, demonstrating a dose-dependent trend, with 41% of participants in the 100 mg twice daily group achieving complete skin clearance at 16 weeks (versus 0% in the placebo group, *p* ≤ 0.05). Adverse events were mild in severity, the most common of which were infections, including COVID-19, nasopharyngitis, and upper respiratory tract infections [42]. JNJ-2113 is currently being investigated in two phase III studies for psoriasis (NCT06095115 and NCT06095102), the latter of which aims to investigate the efficacy and safety of the drug in treating difficult-to-treat areas such as the scalp, palms, soles, and genital regions. A 36-week, phase IIb, long-term extension study (NCT05364554, FRONTIER 2) enrolling participants from the preceding FRONTIER 1 trial was recently completed, however, results have not yet been made available.

## 5. Interleukin-17 Inhibitors

IL-17 is the main effector cytokine in psoriasis [43]. It is produced by Th17 cells, and upon production, acts on keratinocytes, promoting their proliferation, as well as the production of antimicrobial peptides, cytokines, and chemokines that contribute to the formation of psoriasis plaques [44,45,46]. Structurally, the IL-17 family consists of six subunits (IL-17A–IL-17F). IL-17A and IL-17F are particularly implicated in psoriasis signaling, forming two homodimers and the IL-17A/F heterodimer [44,45,46]. Antibodies that inhibit these subunits are successful therapeutically, and oral formulations are currently under investigation [47].

DC-806 (Eli Lilly and Company, Indianapolis, IN, USA) is a small molecule inhibitor of the IL-17A subunit [48]. In a phase I, proof-of-concept study (unlisted) comprising 40 participants, the participants treated with DC-806 800 mg twice daily reported a significantly greater reduction in mean PASI score at 4 weeks compared to those who received the placebo (−43.7% vs. −13.3%; exploratory *p* = 0.0008) [48]. Treatment with low-dose DC-806 (175 mg twice daily) saw no clinically significant benefit. Adverse events were mild-to-moderate in severity, without a dose-dependent trend. Common adverse events were headaches, abdominal discomfort, and COVID-19 infection. No discontinuations due to adverse events or clinically significant changes in lab abnormalities were reported [48]. DC-806 is currently being investigated in a phase IIb dose-ranging trial (NCT05896527), with the PASI 75 response at 12 weeks defined as its primary endpoint. 

IL-17 inhibitors in early phase development include LEO 153339 (LEO Pharma, Ballerup, Denmark), which was recently investigated in a phase I, single ascending dose (SAD) and multiple ascending dose (MAD) trial in healthy subjects (NCT04883333), and DC-853 (Eli Lilly and Company), a fast follower of DC-806. According to Lilly, DC-853 has a higher affinity for the IL-17A cytokine and offers improved metabolic stability [49]. However, no preclinical or clinical trial data on these agents have been published.

## 6. Phosphodiesterase-4 Inhibitors

PDE4 is the main phosphodiesterase expressed in immune cells and keratinocytes [50]. It mediates inflammatory responses by hydrolyzing 3′-5′-cyclic adenosine monophosphate (cAMP), an important second messenger molecule, to adenosine monophosphate (AMP) [51,52,53]. Inhibitors of the PDE4 enzyme are of benefit therapeutically: they raise levels of cAMP intracellularly and activate pathways that support an anti-inflammatory response, inhibiting the production of proinflammatory cytokines and driving the production of anti-inflammatory mediators [51,52,53].

Orismilast (LEO-32731; UNION Therapeutics) is a twice-daily, oral PDE4 inhibitor in clinical development for psoriasis, atopic dermatitis, and hidradenitis suppurativa [54,55,56]. When profiled against a panel of PDEs, orismilast was 2–5 times more potent than apremilast in inhibiting the PDE4 subtype, specifically the PDE4B and PDE4D splice variants, which are linked to inflammation [54]. A modified release formulation of orismilast was investigated in a phase IIb, randomized, dose-finding study (IASOS; NCT05190419), results of which were recently published [56]. The trial enrolled 202 participants with moderate-to-severe plaque psoriasis, and participants were randomized 1:1:1:1 to receive orismilast at 20 mg, 30 mg, or 40 mg or placebo twice daily for 16 weeks. Participants in each treatment group were majority white and male. The primary outcome was met, with participants in all three treatment groups achieving a significantly greater reduction in PASI score at 16 weeks compared to those who received the placebo (−52.6%, −61.2%, −63.7% vs. −17.3%; *p* < 0.001). Significant improvements in PASI 75 and PASI 90 responses were seen across all treatment groups and in the 20 mg and 40 mg groups, respectively. The most common adverse events were diarrhea, headaches, and nausea. The proportion of participants in the 20 mg, 30 mg, and 40 mg groups for which the treatment was discontinued due to treatment-emergent adverse events (TEAEs) was 20.8%, 20.0%, and 39.6%, respectively, highlighting that tolerability may be a limiting factor to treatment, especially at higher doses [56]. There are currently no ongoing trials investigating orismilast in psoriasis. Larger phase III studies are needed to better understand the long-term efficacy, safety, and tolerability of orismilast among patients.

ME3183 is an orally available selective PDE4 inhibitor developed by Meiji Seika Pharma Co. Ltd., Osaka, Japan [57,58]. In preclinical studies, ME3183 was 5- to 40-fold more potent than apremilast in inhibiting inflammatory cytokine production, specifically IL-10, TNF, and interferon (IFN)-γ [57]. The results from a phase II, double-blind, placebo-controlled, randomized trial (NCT05268016) showed that a significantly greater proportion of participants treated with ME3183 once (15 mg) or twice (5 mg, 7.5 mg) daily achieved a 75% reduction in PASI score at 16 weeks compared to those who received the placebo [58]. Participants in the 10 mg, once daily group saw no significant improvement with respect to the primary outcome. Secondary endpoints including PASI 90 and PASI 100 responses favoured the treatment groups at 16 weeks. ME3183 was well tolerated by participants, with the most common adverse events being diarrhea, headaches, and nausea [58]. There are currently no ongoing trials investigating ME3183 in psoriasis.

Mufemilast (Hemay005; Tianjin Hemay Biotech, Tianjin, China) is a highly potent PDE4 inhibitor in phase III development for psoriasis [59]. The agent is being investigated in patients with moderate-to-severe disease at a 60 mg, twice daily dose, with the proportion of participants achieving a PASI 75 response at 16 weeks defined as the trial’s primary endpoint (NCT04839328). Results from previously completed phase I and phase II studies with mufemilast have not yet been published.

## 7. Janus Kinase Inhibitors

Several cytokines in the pathogenesis of psoriasis bind to type I and type II receptors, which rely on the Janus kinase (JAK)–signal transducer and activator of transcription (STAT) pathway for signaling [60,61,62,63,64]. These cytokines include IL-12, IL-23, IL-17, IL-19, and TNF. When a cytokine binds to its receptor, the receptor undergoes a conformational change, recruiting two JAK proteins. The JAK proteins bind and become activated, altering the receptor to facilitate the recruitment of two STAT molecules. Once bound, the STAT molecules phosphorylate, dimerize, and translocate to the nucleus, where they act as transcription factors that alter gene expression [60,61,62,63,64]. 

There are four JAK proteins (JAK1-3 and TYK2) and seven STAT proteins (STAT1, STAT2, STAT3, STAT4, STAT5A, STAT5B, and STAT6) [64]. Different cytokines signal through unique combinations of JAKs [64]. Inhibitors of this pathway are of interest therapeutically and have been developed with varying selectivity [65,66,67]. First-generation agents block multiple JAK proteins, targeting the enzyme’s catalytic site (JH1 domain) and interfering with several downstream signaling pathways. Adverse events, however, remain a concern, with agents in this class failing to gain approval for the disease [66,67]. Efforts to develop more targeted therapies have been the focus of recent research, with agents targeting the TYK2 enzyme seeing the greatest advancement [68,69,70]. In psoriasis, TYK2 plays a key role in disease pathogenesis, mediating IL-12, IL-23, and IFN-α signaling and promoting the development of the Th1 and Th17 cell lines [68,69,70]. Deucravacitinib is the first TYK2 inhibitor to be approved for psoriasis, binding to the enzyme’s allosteric site (JH2 domain) and demonstrating high selectivity against other JAK isoforms [17,71]. Agents with a similar mechanism of action and improved binding potency to the TYK2 enzyme are currently under development, aiming to provide greater efficacy to deucravacitinib with a consistent safety profile. JAK inhibitors that bind to the JH1 domain with greater selectivity are also being studied. 

TYK2 inhibitors in phase II development for psoriasis include ESK-001 (Alumis, Inc., San Francisco, CA, USA) and BMS-986322 (Bristol Myer’s Squib, New York, NY, USA), a structural variant of deucravacitinib (Table 1). In phase I studies, ESK-001 demonstrated potent and selective inhibition of the TYK2 enzyme with no pharmacological inhibition of JAK1-3 (NCT05431634) [72]. When investigated in more than 100 healthy participants, ESK-001 was well tolerated, with no serious adverse events reported [72]. There are currently no available data on BMS-986322 in patients with psoriasis. 

TAK-279 (formerly, NDI-034858) is an allosteric TYK2 inhibitor developed by Takeda Therapeutics [73,74,75,76]. In preclinical trials, TAK-279 was 13.0 × 10^4^ times more selective for the TYK2-JH2 domain than deucravacitinib, owing to a single amino acid difference in its allosteric binding pocket that prevents binding to JAK1 [74,75]. The efficacy and safety of TAK-279 in psoriasis was investigated in a phase II clinical trial (NCT04999839) in which 259 participants were randomized 1:1:1:1:1 to receive TAK-279 at 2 mg, 5 mg, 15 mg, or 30 mg or placebo once daily for 12 weeks [76]. The primary outcome was met, with a significantly greater proportion of patients in all three treatment groups (44%, 68%, and 67% in the 5 mg, 15 mg, and 30 mg groups, respectively) achieving a PASI 75 response at 12 weeks compared to those who received the placebo (6%; *p* < 0.001 for all comparisons). PASI 90 was achieved by 21%, 45%, 46%, and 0% of the participants in the 5 mg, 15 mg, 30 mg, and placebo groups, respectively, and 33% of patients in the 30 mg group reported complete skin clearance (PASI 100 response) at 12 weeks. The most common adverse events were COVID-19 infection, acne, acneiform dermatitis, and diarrhea. Two serious adverse events were reported in the 15 mg group but were considered unrelated to the study drug. No adverse trends in laboratory data (including hematologic, renal, hepatic, and lipid parameters) were reported in any treatment group [76]. Ongoing trials investigating TAK-279 in psoriasis are summarized in Table 1.

TLL-018 (Hangzhou Highlightll Pharmaceutical Co., Ltd., Hangzhou, China) is a highly specific JAK1 and TYK2 inhibitor with IC50 values of 4 nM and 5 nM, respectively [77]. When profiled against a panel of 350 human kinases, TLL-018 was 90-fold more selective for the JAK1 and TYK2 enzymes than all other kinases tested [77]. Like TYK2, JAK1 plays an important role in mediating inflammatory responses, pairing with JAK2, JAK3, or TYK2 to transduce signals from the IFN-α, IFN-γ, and IL-10 receptors [69]. Inhibiting both the TYK2 and JAK1 proteins may provide the benefits of a more efficacious agent while reducing adverse events related with JAK2 and JAK3 inhibition, specifically the risk of thrombotic and cytopenic adverse events as well as the risk of infections, respectively. Data from previously completed phase I trials in psoriasis have not yet been made available. However, results from a phase II trial in rheumatoid arthritis were recently presented and demonstrated positive results with respect to the trial’s primary outcome, with a significantly greater proportion of participants that received TLL-018 at 20 mg and 30 mg twice daily achieving an American College of Rheumatology 50% response at 12 weeks compared to tofacitinib [78]. The most common adverse events were hyperlipidemia and respiratory infections. No deaths, venous thromboembolism, or major adverse cardiovascular events were reported [78]. The efficacy and safety of TLL-018 in psoriasis is currently being investigated in a phase II clinical trial (NCT05772520) in which participants with moderate-to-severe disease will be randomized to receive one of three twice daily doses of TLL-018 or placebo, with the PASI 75 response at 12 weeks defined as the trial’s primary endpoint (Table 1).

Jaktinib (Suzhou Zelgen Biopharmaceuticals) is a twice-daily, selective JAK1/2 inhibitor in phase II development for psoriasis [79]. In a phase I SAD, MAD, and food effect study (NCT03314402), jaktinib was well tolerated by healthy Chinese participants [79]. Adverse events were mild-to-moderate in severity, the most common of which were diarrhea, dizziness, headaches, and neutropenia. One case of grade 3 varicella that was considered treatment-related was reported in the SAD cohort. A dose-dependent trend in neutropenia was observed between groups in the MAD cohort, limiting the maximum tolerated dose to 200 mg once daily [79]. The efficacy and safety of jaktinib in psoriasis is currently being investigated in a phase II clinical trial (NCT04612699) in which participants will receive one of three active doses of jaktinib (50 mg, 75 mg, or 100 mg) or placebo twice daily for 24 weeks. The proportion of participants achieving a PASI 75 response at 12 weeks is defined as the trial’s primary endpoint.

## 8. Sphingosine-1-Phosphate Receptor 1 Agonists 

Sphingosine-1-phosphate (S1P) is a bioactive lipid metabolite that signals through five G protein-coupled receptors (S1PR1-S1PR5) [80,81]. It mediates several cellular processes including cell survival, proliferation, and migration. Specifically, the S1PR1 is expressed on lymphocytes and plays a key role in lymphocyte trafficking, recognizing signals that guide their egress from secondary lymphoid organs to surrounding tissues [82,83,84]. S1PR1 modulators are of interest therapeutically because they promote the internalization of the S1PR1 and prevent lymphocytes from binding to S1P molecules, thus blocking lymphocyte infiltration to sites of inflammation [85,86].

SCD-044 (Vibozilimod; Sun Pharmaceutical Industries Limited) is a S1PR1 agonist in early phase development for psoriasis and atopic dermatitis [87]. According to Sun Pharma, SCD-044 achieved proof of concept in a phase I clinical trial (unlisted), reducing lymphocyte counts for all dose levels evaluated in healthy participants [87]. The drug is currently under investigation in a phase II study (NCT04566666), which aims to investigate the efficacy and safety of three doses of SCD-044 versus placebo in 240 participants with moderate-to-severe plaque psoriasis (Table 1). 

## 9. A_3_ Adenosine Receptor Agonists

The A_3_ adenosine receptor (A_3_AR) is a G_i_-protein coupled receptor found on the surface of peripheral blood mononuclear cells [88]. This receptor is overexpressed in patients with psoriasis, and upon activation, mediates pathways that downregulate NF-κB signaling, reducing TNF expression and promoting an anti-inflammatory response [88,89].

Piclidenoson (CF-101; Can-Fite BioPharma) is an A_3_AR agonist which recently gained approval by the European Medicines Agency and the U.S. Food and Drug Administration (April 2023 and June 2023, respectively) to initiate phase III registration trials for psoriasis [90,91]. The efficacy and safety of piclidenoson has been demonstrated in several previously published phase II and phase III studies [92,93]. Most recently, findings from the phase III COMFORT trial (NCT04168256) were presented [94]. The trial enrolled over 400 participants with moderate-to-severe plaque psoriasis, and participants were randomized 3:3:3:2 to receive one of two doses of piclidenoson (2 mg or 3 mg twice daily), apremilast (30 mg twice daily), or placebo. The primary outcome was met, with a significantly greater proportion of participants in the piclidenoson groups achieving a PASI 75 response at 16 weeks compared to those who received the placebo. Piclidenoson was inferior to apremilast with respect to the proportion of participants achieving a PASI 75 response but was comparable to apremilast with respect to Psoriasis Disability Index (PDI) scores at 32 weeks. Response to piclidenoson improved over the course of the trial, with 90% of participants achieving a PASI 50 response and 10% of participants achieving a PASI 90 response at 48 weeks in the 3 mg group. Piclidenoson was well tolerated by participants, reporting fewer adverse events and trial discontinuations than apremilast and a similar safety profile to placebo [94]. Complete efficacy and safety data from this trial have yet to be published.

## 10. Oral Microbials

Oral microbials are being pursued as a promising therapeutic strategy for psoriasis, with evidence suggesting a link between systemic inflammation and the integrity of the gut microbiome [95,96]. 

EDP1815 is a non-live pharmaceutical preparation of a single strain of the bacterium *Prevotella histicola* developed by Evelo Biosciences [97,98,99,100]. The agent is gut-restricted, non-colonizing, and does not alter the host gut microbiota [97,98,99]. Rather, EDP1815 is believed to interact with innate immune cells in the small intestine, causing them to activate and migrate into nearby mesenteric lymph nodes, communicating with T-lymphocytes and priming them to promote a systemic anti-inflammatory response. This proposed mechanism is supported by data confirming the interaction of EDP1815 with toll-like receptor (TLR) 2 as well as studies demonstrating attenuated anti-inflammatory responses following the blockade of signaling by α4β7 and L-selectin, integrins that mediate dendritic cell migration to mesenteric lymph nodes [97,98,99]. The efficacy of EDP1815 in reducing systemic inflammation has been demonstrated in several preclinical studies in which the drug successfully reduced ear skin thickness in Th1- and Th17-driven mouse models of inflammation [99]. When investigated in a phase I trial for psoriasis, participants treated with high-dose and low-dose EDP1815 saw greater reductions in Lesion Severity Score (LSS) at 4 weeks compared to those who received the placebo with no significant differences in adverse events reported between groups [99]. Most recently, the results from a larger phase II trial were presented (NCT04603027) [100]. The trial enrolled 249 participants with mild-to-moderate psoriasis, and participants were randomized to receive one of three active doses of EDP1815 (1, 4, or 10 tablets) or placebo once daily for 16 weeks. The primary outcome was met, with a significantly greater proportion of participants who received 1 and 4 capsules of EDP1815 achieving a PASI 50 response at 16 weeks compared to those who received the placebo. Following a 24-week optional treatment withdrawal period, 60% of the participants that achieved a PASI 50 score at 16 weeks maintained this level of response with no flare or rebound cases of psoriasis reported. Adverse events were consistent with previously conducted phase I studies, with no serious adverse events or discontinuations due to adverse events observed [100]. There are currently no ongoing trials investigating EDP1815 for psoriasis.

KBL697 is a probiotic strain of the bacterium *Lactobacillus gasseri* developed by KoBioLabs [101]. Unlike EDP1815, this agent is live and alters the gut microbiome. The use of probiotics in psoriasis may be supported by previous studies that have demonstrated a reduction in the diversity of intestinal microbiota in the stool samples of patients with psoriasis compared to those of normal controls [95,96]. This imbalance of certain commensal bacteria, termed intestinal dysbiosis, may trigger systemic inflammatory responses, leading to a disruption of cutaneous homeostasis [95,96]. The efficacy and safety of high-dose versus low-dose KBL697 is currently being investigated in a phase II clinical trial for psoriasis (NCT04911751). Results from a previously completed phase I SAD and MAD study have not yet been made available (NCT04056130).

## 11. Conclusions

The ideal psoriasis therapy is one that is cost-effective and offers ease of use, efficacy, and safety with minimal adverse events. As our understanding of the pathogenesis of psoriasis has evolved in recent decades, biologic agents have seen the greatest advancement with several new agents approved for the disease. Oral therapies, however, have been slower to follow suit despite these agents being more cost-effective and generally favoured by patients. Oral agents with well-validated and new mechanisms of action are currently under development for psoriasis. JNJ-2113, an agent targeting the IL-23 receptor, recently initiated phase III trials for the disease following promising results in phase II studies. Orismilast, a selective PDE4 inhibitor, was successful in reducing disease severity in phase II trials, however, adverse gastrointestinal events may be a limiting factor and larger trials are needed to better discern its safety and tolerability profiles. New JAK inhibitors are also being studied with greater potency for the TYK2 allosteric site. A_3_AR agonists and novel oral microbial agents show promise in offering new classes of therapies for patients with psoriasis. 

As new oral therapies are welcomed to the treatment landscape for psoriasis, long-term trials and comparative studies with existing oral and biologic agents are needed to better understand their place in the current treatment algorithm. Agent-specific characteristics, including tolerability and dosing regimen are also important to recognize with several medications requiring twice daily dosing schedules that should be considered based on potential issues with patient compliance. Studies investigating oral therapies in patients with comorbidities for psoriasis will also be necessary to better inform optimal selection for patients.

## Figures and Tables

**Table 1:** Reviewed pipeline oral therapies for psoriasis.

Name	MOA	Highest Phase	Ongoing Trials in Psoriasis	Primary Endpoint	Comments
SAR441566	TNF	Phase 2	NCT06073119	-PASI 75 at 12 weeks	
JNJ-2113	IL-23 receptor	Phase 3	NCT06095115	-IGA score of 0/1 + a >/= 2-grade improvement from baseline at 16 weeks -PASI 90 at 16 weeks	-Phase III trial enrolling adolescents and adults with moderate-to-severe plaque psoriasis (ICONIC-LEAD); dosing regimen undisclosed
			NCT06095102	-IGA score of 0/1 + a >/= 2-grade improvement from baseline at 16 weeks	-Phase III trial investigating the efficacy and safety of JNJ-2113 in treating special areas of the skin in patients with psoriasis, including the scalp, palms, soles, and genital areas (ICONIC-TOTAL)
DC-806	IL-17A	Phase 2	NCT05896527	-PASI 75 at 12 weeks -Incidence of TEAEs, SAEs, and TEAEs leading to discontinuation up to 16 weeks, 20 weeks, and 16 weeks, respectively	
LEO 153339	IL-17A	Phase 1	-	-	-Phase 1 SAD and MAD study completed in July 2022 (NCT04883333); trial enrolled healthy adult participants
DC-853	IL-17A	Phase 1	-	-	-Currently in phase I development; trial unlisted
Orismilast	PDE4	Phase 2	-	-	-Phase II trial (NCT05190419) completed in December 2022; trial investigated three twice daily (20 mg, 30 mg, and 40 mg) doses of a modified-release formulation of orismilast versus placebo for 16 weeks in patients with moderate-to-severe plaque psoriasis
ME3183	PDE4	Phase 2	-	-	-Phase IIa trial (NCT05268016) completed in May 2023 that investigated two once daily and two twice daily doses of ME3183 versus placebo in patients with psoriasis for 16 weeks; primary endpoint defined as the PASI 75 response at 16 weeks
Mufemilast	PDE4	Phase 3	NCT04839328	-PASI 75 at 16 weeks	
ESK-001	TYK2	Phase 2	NCT05739435	-Incidence of TEAEs and SAEs up to 3 years	-Long-term extension study enrolling participants from the preceding phase II trial (NCT05600036); participants to receive one of two open-label doses of ESK-001 for 3 years
BMS-986322	TYK2	Phase 2	NCT05730725	-PASI 75 at 12 weeks -Multiple safety measures (incidence of SAEs, TEAEs, laboratory abnormalities, etc., up to 16 weeks)	
TAK-279	TYK2	Phase 3	NCT06088043	-sPGA score of 0/1 + a >/= 2-grade improvement from baseline at 16 weeks -PASI 75 at 16 weeks	-Head-to-head trial investigating TAK-279 versus apremilast and placebo in patients with moderate-to-severe plaque psoriasis; treatment period of 52 weeks; trial to enroll 600 participants
			NCT06108544	-sPGA score of 0/1 + a >/= 2-grade improvement from baseline at 16 weeks -PASI 75 at 16 weeks	-Head-to-head trial investigating TAK-279 versus apremilast and placebo in patients with moderate-to-severe plaque psoriasis; 60-week follow-up period, including a withdrawal and retreatment period; trial to enroll 1000 participants
TLL-018	JAK1/TYK2	Phase 2	NCT05772520	-PASI 75 at 12 weeks	
Jaktinib	JAK1/JAK2	Phase 2	NCT04612699	-PASI 75 at 12 weeks	
SCD-044	S1PR1	Phase 2	NCT04566666	-PASI 75 at 16 weeks	-Phase II trial (SOLARES-PsO-1) including a 16-week, double-blind period and a 36-week, long-term extension period; participants to receive one of three doses of SCD-044 or placebo; dosing regimen undisclosed
Piclidenoson	A_3_AR	Phase 3	-	-	-Results from a previously completed phase III trial (NCT03168256) submitted in August 2023
EDP1815	*Prevotella histicola*	Phase 2	-	-	-Phase II trial for psoriasis; results were posted in December 2022 (NCT04603027)
KBL697	*Lactobacillus gasseri*	Phase 2	NCT04911751	-Mean change in PASI score from baseline to 12 weeks	

MOA = mechanism of action, TNF = tumor necrosis factor, PASI = Psoriasis Area and Severity Index, IL = interleukin, SAD = single ascending dose, MAD = multiple ascending dose, IGA = Investigator’s Global Assessment, TEAEs = treatment-emergent adverse events, SAE = serious adverse events, PDE = phosphodiesterase, TYK = tyrosine kinase, JAK = Janus kinase, S1PR1 = sphingosine-1-phosphate receptor 1, sPGA = Static Physician’s Global Assessment, A_3_AR = A3 adenosine receptor.
